# Rare case of concomitant juxtarenal aortic aneurysm and type 1 Inferior Vena Cava duplication: -intraoperative challenges to avoid catastrophe

**DOI:** 10.1590/1677-5449.200155

**Published:** 2021-12-10

**Authors:** Cherring Tandup, Arunanshu Behera, Kailash Chand Kurdia, Charan Singh

**Affiliations:** 1 Post Graduate Institute of Medical Education & Research – PGIMER, Chandigarh, India.

**Keywords:** abdominal aortic aneurysm, IVC anomaly, aneurisma de aorta abdominal justarrenal, anomalia VCI

## Abstract

Duplication of the inferior vena cava is a rare congenital anomaly, with an incidence of 0.2-3%. Despite being asymptomatic, anomalies of IVC are important in aortoiliac and retroperitoneal surgeries. Preoperative CT imaging is essential to identify any IVC anomaly and to prevent unexpected hemorrhage during surgery. Here, we report a case of a juxtarenal abdominal aortic aneurysm in which we encountered a type I IVC duplication anomaly intraoperatively while performing transperitoneal aneurysmorrhaphy and took precautions to avoid any iatrogenic injuries to either of the two trunks or the pre-aortic trunk of the anomalous duplicate IVC.

## Resumo

A duplicação da veia cava inferior (VCI) é uma anomalia congênita rara com incidência de 0,2 a 3%. Apesar de assintomáticas, anomalias da VCI são importantes em cirurgias aortoilíacas e retroperitoneais. A imagem da tomografia pré-operatória é essencial para identificar qualquer anomalia de VCI e para evitar hemorragia inesperada durante a cirurgia. Relatamos um caso de aneurisma de aorta abdominal justarrenal, no qual encontramos uma anomalia de duplicação de VCI do tipo 1 intraoperatório enquanto realizávamos correção cirúrgica de aneurisma transperitoneal. Por isso, tomamos a precaução para evitar qualquer lesão iatrogênica nos dois troncos e no tronco pré-aórtico de VCI duplicada anômala.

## INTRODUCTION

Although uncommon, anomalies of the inferior vena cava have important implications for the vascular surgeon. The presence of inferior vena cava anomalies can create technical difficulties during aortoiliac surgeries or retroperitoneal surgeries and injury to an unrecognized anomalous vein can result in unexpected severe hemorrhage. Identification of these anomalies and appropriate treatment are therefore of the utmost importance.^1^

Congenital venous abnormalities in the retroperitoneal space are rare and usually asymptomatic. There are four major variations of the venous system in the retroperitoneum.^2^ These anomalies have low prevalence rates, as follows: retroaortic left renal vein (LRV), with an incidence of 0.3–0.9%; circumaortic LRV (0.5–1.4%); IVC duplication (0.2–3%); and left-sided IVC (0.2–0.5%).^2^ These anomalous veins are typically dilated, thin-walled, and tortuous and are more prone to injury during abdominal aortic surgery.

## CASE REPORT

A 55-year-old female was referred to our institution with a diagnosis of juxtarenal abdominal aortic aneurysm. She had complaints of severe abdominal pain over the past 10 days, which was radiating to the back and was also associated with heaviness over the entire abdomen. On examination, tachycardia was present and she was hypertensive with blood pressure of around 150/96mmhg, with mild pallor. Abdominal examination revealed fullness over the epigastric region, which was pulsatile. We avoided palpation.

**Figure 1:**
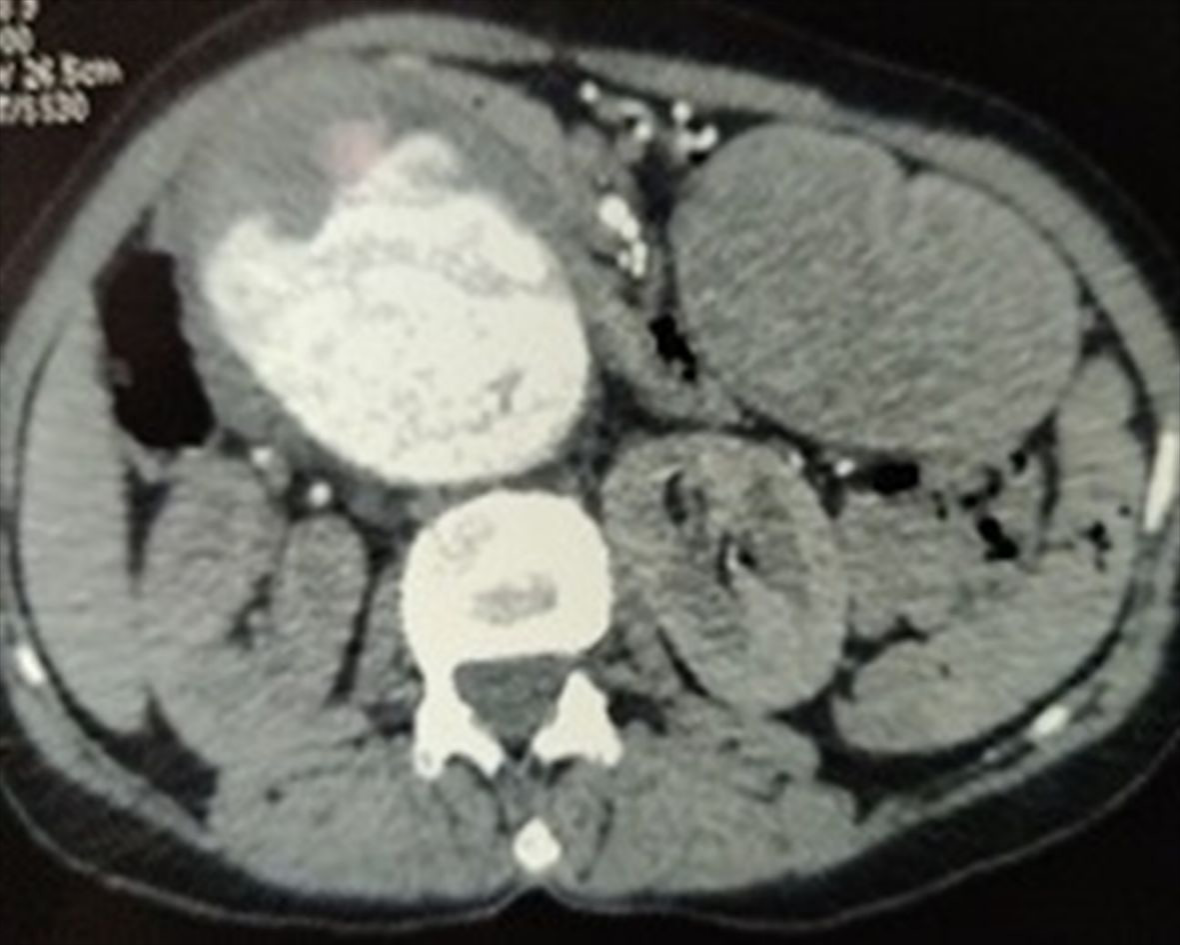
CT angiography of abdominal aorta revealing a large fusiform aneurysm of the abdominal aorta extending from D12 to L5 vertebral bodies, to the bifurcation, with eccentric, irregular, and largely non-calcified thrombus along the right lateral wall.

**Figure 2:**
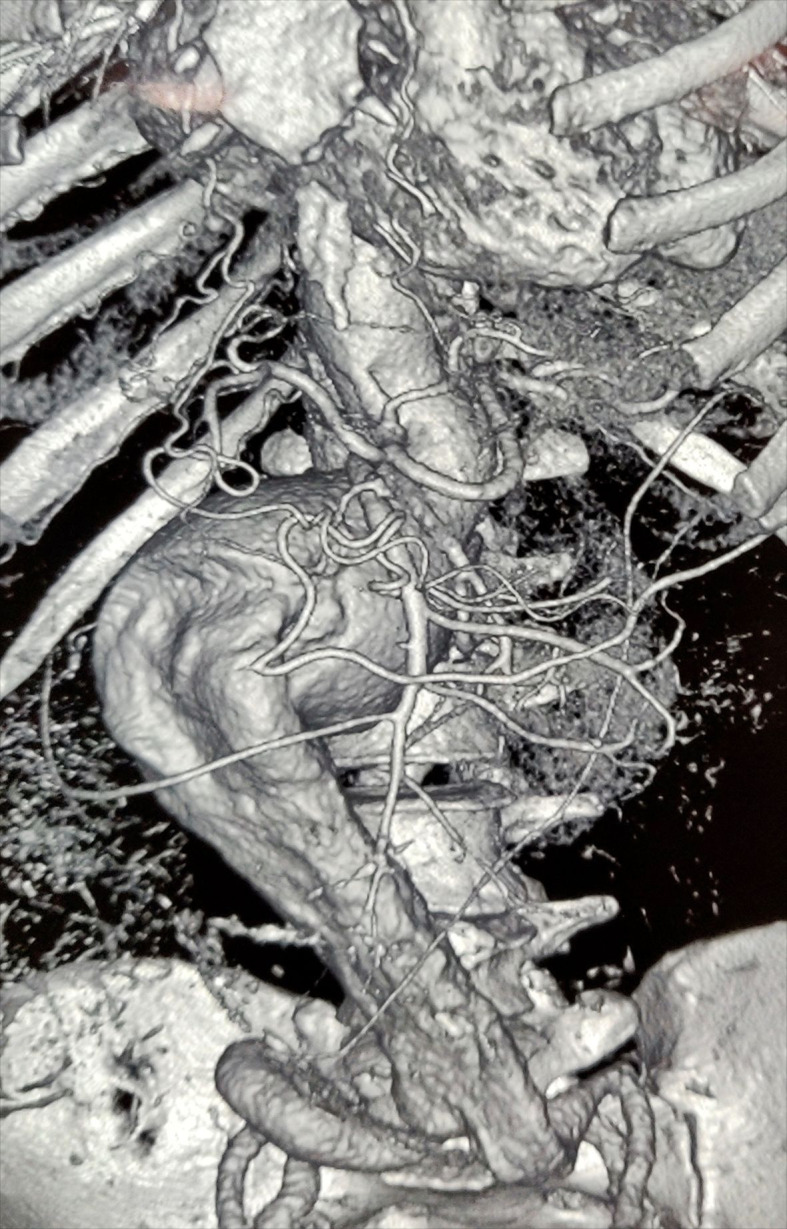
Three-dimensional reconstruction image of abdominal aorta along with bilateral common iliac artery showing large fusiform saccular aneurysm of the abdominal aorta measuring 17.7 cm in length and extending from D12 to L5 vertebral bodies, to the bifurcation, with a neck diameter of 2.5 cm.

On CT Angiography (Figures 1 and 2) a large fusiform saccular aneurysm was present in the abdominal aorta, measuring 17.7 cm in length and extending from D12 to L5 vertebral bodies, to the bifurcation, and eccentric, irregular, and largely non-calcified thrombi were seen along the right lateral walls of the aneurysm, which had a neck diameter of 2.5 cm. We scheduled her for open transperitoneal aneurysmorrhaphy. She was started on anti-hypertensive metoprolol 100 mg which was later reduced to 50 mg and telmisartan 40 mg was also started, targeting systolic pressure of <130 mmHg. Preoperative CT images did not reveal duplication of the IVC, probably due to IVC compression because of the large aortic aneurysm. EVAR was not feasible due to technical issues.

**Figure 3:**
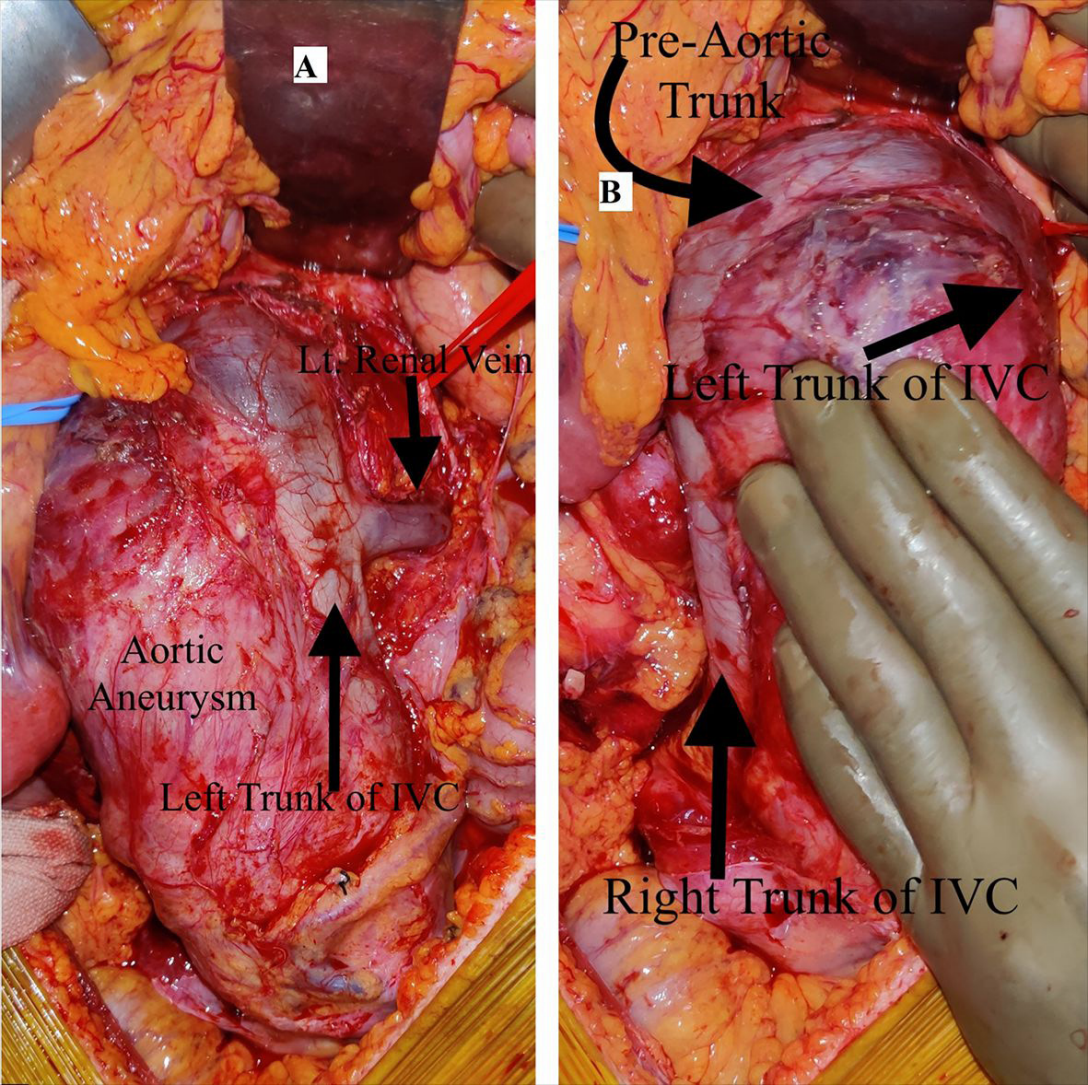
(**A**) Intraoperative image showing left trunk of Inferior Vena Cava continuing as preaortic trunk. The large abdominal aortic aneurysm can be observed; (**B**) Intraoperative image showing abdominal aortic aneurysm along with Type 1 IVC duplication anomaly.

A standard midline approach was used and a large saccular juxtarenal aortic aneurysm with dimensions of 18 cm x 7cm was noted. The transverse colon was retracted cephalad, and the small bowel loops were wrapped in a wet sheet and retracted to the left side, as opposed to the conventional approach. We carefully dissected the neck of the aneurysm, retracing the left renal vein to take proximal control just above the insertion of the renal artery. During dissection, we identified a large diameter vein coursing along the left side of the aneurysm sac (Figure 3) which we then followed to find a preaortic trunk continuing to the right-side IVC. Thus, an intraoperative finding of type 1 IVC duplication was made, which was very much essential, since if we would have missed it then injury to the preaortic trunk during opening of aneurysm sac could have led to torrential bleeding. Suprarenal clamping space was created and bilateral renal arteries were looped. After heparinization, the clamp was applied above the bilateral renal arteries and we packed the kidneys on both sides with cold saline solution for renal hypothermia. A 22 x 11 mm knitted graft was used for aneurysm repair, the proximal anastomosis was completed in 20 minutes, and renal reperfusion was maintained. Distal anastomosis was done to common iliac arteries bilaterally. Postoperatively, the patient did well. She developed ileus, which was resolved medically, and is in follow up.

For case report publication we don’t require approval from Institutions ethic committee. Only consent from the patient for his discussion in the case report.

## DISCUSSION

The inferior vena cava is formed by right and left common iliac veins at the level of the fifth lumbar vertebra. The abdominal part of the IVC is right-sided in nearly 98% of people and brings blood to the right atrium from below the diaphragm.^3^ The IVC develops in a complex process, which starts in the fourth week of gestation and is completed by the eighth week. There is development, regression, anastomosis, and replacement of three pairs of embryonic veins (the posterior cardinal, subcardinal, and supracardinal veins).^4^ The posterior cardinal veins appear first and regress, except for the distal aspects, which become the iliac bifurcation. The subcardinal veins appear in the fifth week and form the stem of the left renal vein, the suprarenal (adrenal) veins, and the prerenal segment of the IVC. The left subcardinal vein completely regresses. Subsequently, the supracardinal veins develop. The left regresses and the right forms the infrarenal IVC. Persistence of the left supracardinal vein is the cause of most anomalies of the IVC.^5^

Despite the asymptomatic nature of IVC anomalies, their identification is important in aortic and retroperitoneal surgeries. In our case, we performed open repair of a juxtarenal aortic aneurysm and the presence of IVC duplication was identified intraoperatively. Preoperative identification on CT imaging is crucial for planning appropriate repair of an aortic aneurysm to avoid injury to the anomalous vein and avoid unexpected hemorrhage. IVC duplication can be further classified into three types. Type I (or major duplication), which was present in our case, consists of two bilaterally symmetrical and same caliber trunks and a preaortic trunk of the same caliber. Type II comprises two bilaterally symmetrical and same caliber trunks, but smaller than the preaortic trunk, and type III is the asymmetric trunk which comprises small left IVC and a large right IVC, with an even larger preaortic trunk, or may be a small left IVC then the large preaortic trunk, with a larger right IVC.

## CONCLUSION

It is important to rule out any venous malformation preoperatively in any aortoiliac surgery or retroperitoneal surgery to prevent iatrogenic hemorrhage due to IVC injury, because such vessels have thin walls and are dilated and tortuous. Even if preoperative imaging does not show any anomaly, knowledge of such anomalies should be kept in mind while operating to prevent hemorrhage. Surgical techniques must be modified with careful proximal clamping of the aorta and opening of sac to avoid injury to the anomalous vein. Presence of IVC anomaly should be dealt with meticulously during surgery to avoid any iatrogenic injury.
